# Supplementary material for: The Antitumoral/Antimetastatic Action of the Flavonoid Brachydin A in Metastatic Prostate Tumor Spheroids In Vitro Is Mediated by (Parthanatos) PARP-Related Cell Death
Source: Pharmaceutics. 2022 Apr 29;14(5):963. doi: 10.3390/pharmaceutics14050963 (PMC9147598; doi:10.3390/pharmaceutics14050963)
Supplement: Supplementary file 1 [file pharmaceutics-14-00963-s001.zip › pharmaceutics-1668907-supplementary.pdf]

pharmaceutics

Supplementary Material

Materials and Methods

Comet assay (Single Cell Gel Electrophoresis)

The genotoxicity assay was performed using the protocol reported by Olive et al.[69], with modifications, and following the recommendations from Moller [70]. After the initiation step, DU145 spheroids were treated for 4 h with RPMI 1640 (NC), 1% DMSO (SC), 50 μM DTX (PC), or BrA (40 - 100 μM). Next, six spheroids were transferred to a 1.5 mL microtube, centrifuged for 5 min at 300 ×g, and treated with TrypLE™ enzyme 1X for 5 min, at 37 °C, in a water bath, and manually shaken every 1 min. Cell viability was analyzed with Trypan blue (0.4%) staining using Countess™ Automated Cell Counter (Thermo Fischer Scientific). All the samples with cell viability ≥ 80% were submitted to the next steps. Then, the resulting pellet was suspended in PBS and homogenized with 0.5% low melting point (LMP) agarose (Gibco) (1:4 μL homogenate/LMP agarose; v/v); this mixture was transferred to a conventional slide pre-coated with 1.5% NMP agarose (Gibco) and covered with a coverslip (24 x 60 mm). The slides were incubated for 10 min at 4 °C for agarose solidification. The coverslips were removed, and the slides were immersed in a lysis solution (2.5M NaCl, 100 mM EDTA, 10 mM Tris, 10% DMSO, 1% Triton X-100, pH 10) for 20-22 h at 4 °C. Then, the slides were immersed in alkaline electrophoresis buffer (300 mM NaOH, 1 mM EDTA, pH > 13) for 20 min. The electrophoresis was performed at 25 V and 300 mA (0.74 V/cm1) for 20 min, using the same alkaline electrophoresis buffer. All steps were carried out without the light incidence. Next, the slides were immersed in neutralization solution (0.4 M Tris, pH 7.5 at 4 ° C) for 5 min, dried at room temperature, and fixed in ethanol 99% (Sigma-Aldrich) for 5 min. At the time of analysis, the slides were stained with GelRedTM (1:1000 μL Gel Red:PBS; v/v) (Biotium, FR, USA) and examined in a fluorescence microscope (Zeiss-AxioStar Plus) set at 515-560 nm with a 590 nm barrier filter, using a 20x objective. For each culture, 100 nucleoids were analyzed (300 nucleoids per treatment) with the CometAssay IV software (Perceptive; Suffolk, England); the tail intensity (% DNA in the tail) was the parameter analyzed. All analyses were performed in three biological experiments (n = 3).

Results

Docetaxel is cytotoxic to metastatic DU145 prostate spheroids

The chemotherapy drug Docetaxel (DTX) was selected as the positive control and tested at concentrations of 1, 5, 10, and 50 μM in DU145 spheroids at the treatment times of 24, 48 and 72 h. DTX at concentrations ≥ 10 μM decreased cell viability of DU145 spheroids after 48 and 72 h of treatment (Table S1). Then, the DTX concentration of 50 μM was selected for the subsequent biological assays.

Table S1. Cell viability (%) of DU145 spheroids after treatments with docetaxel (DTX) for 24, 48 and 72 h of treatment, as assessed using the resazurin assay[1].

Exposure Time (h)	NC	Docetaxel (μM) 1	5	10	50
24	101.0 ± 1.0	97.2 ± 1.7	99.9 ± 3.4	89.5 ± 3.8	75.7 ± 7.8*
48	101.7 ± 2.1	95.3 ± 2.0	100.6 ± 1.0	90.1 ± 2.8*	84.6 ± 3.9*

Exposure Time (h)	NC	Docetaxel (μM) 1	5	10	50
72	101.3 ± 2.3	96.2 ± 1.2	97.6 ± 1.1	91.9 ± 4.5*	76.7 ± 2.1*

[1]All values are presented as the mean ± standard deviation (X ± SD) of six spheroids/replicates (n = 6) in three biological experiments (n = 3). *Values statistically different from NC at the respective time point (day) (*p < 0.05; ANOVA followed by Dunnett's post-test). NC: Negative control (RPMI 1640); DTX: Docetaxel.

Short treatment with BrA does not induce genotoxicity in DU145 spheroids

Treatment with BrA (40 - 100 μM) for 4 h did not promote genotoxicity in DU145 spheroids, as assessed using the alkaline comet assay (Figure S1). Our data indicated that BrA did not induce cytotoxicity and cell death due to DNA damage induction.

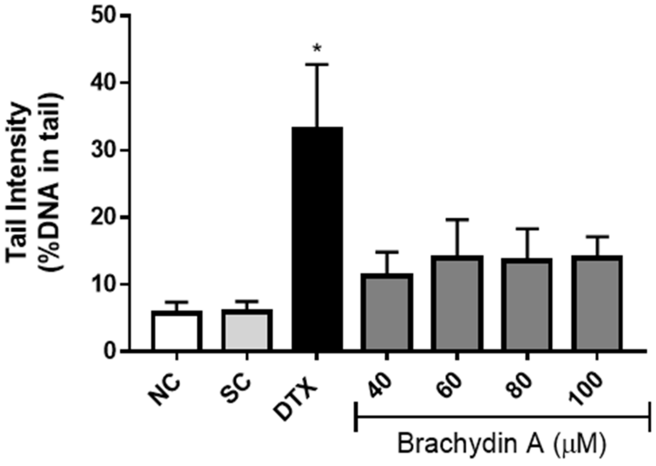

Figure S1. Percentage (%) of DNA in the tail (Tail Intensity) of 300 nucleoids (100 per replicate) in DU145 spheroids treated with BrA for 4 h and the respective controls, as assessed using the alkaline comet assay. The bars represent the mean ± standard deviation of experiments with six (n = 6) spheroids/replicates and three biological experiments (n = 3). *Values statistically different from the NC group (p < 0.05; ANOVA followed by Dunnett's post-test). NC: Negative control (RPMI 1640); SC: Solvent control (1% DMSO); DTX: Docetaxel (50 μM; positive control).

References

69. Olive, P.L.; Vikse, C.M.; Banath, J.P. Use of the comet assay to identify cells sensitive to tirapazamine in multicell spheroids and tumors in mice. *Cancer Res* **1996**, *56*, 4460-4463.

70. Møller, P.; Azqueta, A.; Boutet-Robinet, E.; Koppen, G.; Bonassi, S.; Milić, M.; Gajski, G.; Costa, S.; Teixeira, J.P.; Costa Pereira, C.; et al. Minimum Information for Reporting on the Comet Assay (MIRCA): recommendations for describing comet assay procedures and results. *Nat Protoc* **2020**, *15*, 3817-3826, doi:10.1038/s41596-020-0398-1.
